# Capsule networks for segmentation of small intravascular ultrasound image datasets

**DOI:** 10.1007/s11548-021-02417-x

**Published:** 2021-06-14

**Authors:** Lennart Bargsten, Silas Raschka, Alexander Schlaefer

**Affiliations:** Hamburg University of Technology, Institute of Medical Technology and Intelligent Systems, Hamburg, Germany

**Keywords:** Deep learning, Capsule networks, Intravascular ultrasound, Small datasets, Image segmentation

## Abstract

**Purpose:**

Intravascular ultrasound (IVUS) imaging is crucial for planning and performing percutaneous coronary interventions. Automatic segmentation of lumen and vessel wall in IVUS images can thus help streamlining the clinical workflow. State-of-the-art results in image segmentation are achieved with data-driven methods like convolutional neural networks (CNNs). These need large amounts of training data to perform sufficiently well but medical image datasets are often rather small. A possibility to overcome this problem is exploiting alternative network architectures like capsule networks.

**Methods:**

We systematically investigated different capsule network architecture variants and optimized the performance on IVUS image segmentation. We then compared our capsule network with corresponding CNNs under varying amounts of training images and network parameters.

**Results:**

Contrary to previous works, our capsule network performs best when doubling the number of capsule types after each downsampling stage, analogous to typical increase rates of feature maps in CNNs. Maximum improvements compared to the baseline CNNs are 20.6% in terms of the Dice coefficient and 87.2% in terms of the average Hausdorff distance.

**Conclusion:**

Capsule networks are promising candidates when it comes to segmentation of small IVUS image datasets. We therefore assume that this also holds for ultrasound images in general. A reasonable next step would be the investigation of capsule networks for few- or even single-shot learning tasks.

## Introduction

Intravascular ultrasound (IVUS) is a commonly used imaging modality worldwide. Via IVUS experienced, physicians can assess vessel morphologies and thereby estimate important shape parameters like lumen diameter, vessel wall thickness or plaque burden. This effectively improves treatment planning and thus the success of percutaneous coronary interventions [21].

In order to derive vessel shape parameters from IVUS, physicians have to manually delineate the respective structures in multiple images. This procedure is rather time-consuming, and the results depend strongly on the physicians’ experience. Automatic segmentation of lumen and vessel wall can streamline the derivation of meaningful vessel parameters and therefore improve the efficiency of respective clinical workflows.

Automatic segmentation of lumen and vessel wall via non-data-driven methods has been studied before [1, 13, 14, 23, 27]. Many of these approaches rely on active contour models, level sets, gradient-based techniques or thresholding. For example, in [27], the authors propose a fuzzy clustering approach with superpixels for reducing the influence of speckle noise, followed by a level set evolution algorithm with a new edge indicator. Reviews regarding IVUS segmentation approaches can be found in [1, 13]. Data-driven methods include support vector machines, random forests or convolutional neural networks (CNNs). The authors of [4], e.g., combine an ensemble support vector machine pixelwise classifier with a deformable model to extract lumen and media-adventitia borders. Approaches using CNNs mainly rely on encoder–decoder architectures like U-Net [20] and report state-of-the-art results for segmentation of lumen and vessel wall [7, 15, 17, 19, 28, 29, 31]. However, CNNs depend heavily on the size of the underlying dataset as well as the quality of the corresponding annotations. To ensure high quality, annotations have to be created by trained experts in a time-consuming process which generally leads to rather small datasets in the medical domain. Therefore, it is essential to develop methods which also perform well and robustly on small datasets.

Possible directions to achieve this are incorporating domain knowledge into the CNN [2] or exploiting new sophisticated network architectures. Such a rather novel network architecture is the capsule network [9, 22]. Capsules are neurons grouped into tensors, like vectors or matrices, which correspond to entities and their respective properties (e.g., pose, texture, deformation, etc.) present in the image. These capsules form the basic network elements instead of single neurons as in the case of CNNs. An iterative routing algorithm couples child capsules to parent capsules which thus form a part-whole relationship. The overall network can therefore be interpreted as some kind of parse tree.

Recent experimental studies showed that capsule networks can outperform CNNs when dealing with small natural image datasets [11, 12, 30]. We study whether this also holds for small ultrasound image datasets. We consider the task of segmenting lumen and vessel wall in IVUS images. So far, capsule networks have been applied to X-ray as well as computed tomography image segmentation. Ultrasound images differ a lot from the former modalities regarding texture and noise structure (speckle). Therefore, we assume that the capsule network architecture has to be tuned in order to achieve sufficient segmentation performance on ultrasound images. Our contribution is twofold. First, we present an optimized capsule network for IVUS image segmentation. Second, we provide a detailed analysis of capsule networks and a state-of-the-art CNN with respect to the amount of training data available.

## Material and methods

### Dataset

For this study, we used a publicly available IVUS segmentation dataset consisting of 435 annotated IVUS frames with a size of 384 $\times$ 384 pixels obtained from ten different patients [1]. The images were acquired in a gated fashion with a 20 MHz phased array transducer and annotated by clinical experts by delineating the lumen border and the external elastic membrane as the transition between media and adventitia. The contours were transformed into pixel masks comprising three classes: lumen, vessel wall (as the union of intima and media) as well as background (adventitia and surrounding tissue). See Fig. 3 for exemplary images with corresponding segmentation contours (yellow dashed lines).

In addition, we used another IVUS dataset also provided by [1]. This dataset comprises 77 images from 22 patients with a size of 512 $\times$ 512 pixels. The images were acquired with a rotational transducer and a frequency of 40 MHz. Analogous to the other dataset, the annotations delineate lumen border and external elastic membrane. However, these are much less visible compared to the 20 MHz dataset and thus generally harder to detect (see Fig. 4).

### Capsule networks

Capsules have been developed in order to integrate parse tree-like child–parent relationships into neural networks. Capsules are groups of multiple neurons and can have different forms like vectors [22] or matrices [10]. The general idea is that an active capsule represents a specific entity present in the image, whereas the activities of the corresponding neurons encode its properties like pose, texture or deformation. Capsules in subsequent layers are coupled via an iterative routing process which ensures a part-whole tree structure throughout the network. This means that capsules $\mathbf {u}_i$ in layer *L* (child capsules) with a strong coupling to specific capsules $\mathbf {v}_j$ in layer $L+1$ (parent capsules) can be interpreted as parts of entities represented by the respective parent capsules. To perform the routing procedure, child capsules are transformed into the parent capsules’ feature space via transformation matrices $\mathbf {W}_{ij}$ which are learned via backpropagation.

Since each image entity is associated with a capsule, the activation of a capsule is independent of the entity’s pose. Therefore, capsule layers are—at least heuristically—equivariant [10]. Not only in the case of translations, as CNNs, but also for more complex transformations like rotations or reflections. This could be a reason why capsule networks can outperform CNNs when trained with small datasets as shown in [11, 12, 30].

Considering the case of capsules with vector outputs, the transformation of child capsule output vectors $\mathbf {u}_i$ into the parent capsules’ feature space can be written as

$$\begin{aligned} \hat{\mathbf {u}}_{j|i} = \mathbf {W}_{ij}\,\mathbf {u}_i. \end{aligned}$$

The transformed child capsule output vectors $\hat{\mathbf {u}}_{j|i}$ are linearly combined with weights $c_{ij}$, which are derived from the dynamic routing process in every forward pass (see [22] for details):

$$\begin{aligned} \mathbf {s}_j = \sum _i c_{ij}\,\hat{\mathbf {u}}_{j|i}. \end{aligned}$$

Finally, the parent capsule outputs $\mathbf {v}_j$ are computed via the *squash* activation function:

$$\begin{aligned} \mathbf {v}_j = \text {squash}(\mathbf {s}_j) = \frac{\Vert \mathbf {s}_j\Vert ^2}{1 + \Vert \mathbf {s}_j\Vert ^2}\,\frac{\mathbf {s}_j}{\Vert \mathbf {s}_j\Vert }. \end{aligned}$$

By learning a reverse-encoding of object properties, capsule networks provide improved generalizability to unseen transformations and viewpoint changes while requiring less training data than CNNs when performing pose prediction [10]. Furthermore, the preservation of spatial part-whole relationships can better represent constraints regarding anatomical information which could be quite beneficial for semantic segmentation tasks [22].

The first attempt of using capsule networks for image segmentation was SegCaps [16]. SegCaps introduced locally constrained dynamic routing, which restricts the set of child capsules routed to a specific parent capsule to a relatively small window of size 5 $\times$ 5, analogous to the convolutional kernel size in CNNs. We refer to this type of layer as *convolutional capsule layer*. Furthermore, SegCaps makes use of shared transformation matrices for capsules inside these specific windows. The basic architecture follows a U-Net-like structure incorporating downsampling and upsampling via strided routing windows and skip connections between the encoding and decoding path. The numbers of capsule types—as an analogue to feature maps in CNNs—after each level of the encoding path are {1,4,8,8}. We refer to this expression as the *shape* of the network, because the decoding path usually exhibits the same structure but vice versa.

In contrast to SegCaps, Matwo-CapsNet [3] consists of capsules represented as matrices as proposed by Hinton et al. [10]. Matwo-CapsNet extends the idea of a 4 $\times$ 4 capsule pose matrix by introducing an additional 5 $\times$ 5 appearance matrix and a dual routing algorithm combining the information from both matrices. The term pose matrix should not indicate that this matrix has specific properties which hold for pose matrices in robotics and navigation. Like SegCaps, Matwo-CapsNet exhibits a U-Net-like architecture with convolutional capsule layers and a shape of {5,5,6,7}, whereas the decoding path has six capsule types instead of five.

The forward propagation in Matwo-CapsNet works basically the same as when using vector capsules. Pose matrices $\mathbf {P}_{i}$ and appearance matrices $\mathbf {A}_{i}$ of layer *L* are transformed via transformation matrices $\mathbf {W}^P_{ij}$ and $\mathbf {W}^A_{ij}$:

$$\begin{aligned} \hat{\mathbf {P}}_{j|i} = \mathbf {P}_{i}\,\mathbf {W}^P_{ij} \qquad \hat{\mathbf {A}}_{j|i} = (\mathbf {A}_{i} + b_{ij})\,\mathbf {W}^A_{ij}, \end{aligned}$$

where $b_{ij}$ denotes learnable biases. The transformed matrices $\hat{\mathbf {P}}_{j|i}$ and $\hat{\mathbf {A}}_{j|i}$ are linearly combined with weights $c_{ij}$ which are the same for both types of matrices.

$$\begin{aligned} \tilde{\mathbf {P}}_j = \sum _i c_{ij}\,\hat{\mathbf {P}}_{j|i} \qquad \tilde{\mathbf {A}}_j = \sum _i c_{ij}\,\hat{\mathbf {A}}_{j|i} \end{aligned}$$

The weights are derived from the dual routing procedure (see [3] for details). The output matrices of layer $L+1$ are then calculated by applying the nonlinear activation functions *Psquash* and *squash*.

$$\begin{aligned} \mathbf {P}_j&= \text {Psquash}(\tilde{\mathbf {P}}_j) = \frac{\tilde{\mathbf {P}}_j}{\max (\text {abs}(\tilde{\mathbf {P}}_j))},\\[2mm] \mathbf {A}_j&= \text {squash}(\tilde{\mathbf {A}}_j) = \frac{\Vert \tilde{\mathbf {A}}_j\Vert ^2}{1 + \Vert \tilde{\mathbf {A}}_j\Vert ^2}\,\frac{\tilde{\mathbf {A}}_j}{\Vert \tilde{\mathbf {A}}_j\Vert }. \end{aligned}$$

Capsule networks offer the possibility of incorporating a regularization by performing a reconstruction of the input image from the network’s last capsule layer. In the case of classification, this can be accomplished by feeding the active capsule from the classification layer into a decoder network [22]. In the case of binary segmentation, SegCaps masks out all capsules of the last network layer which do not belong to the target class and feeds the remaining capsules into a decoder consisting of three 1 $\times$ 1 convolutional layers. Matwo-CapsNet waives the idea of a regularization via reconstruction.

### Optimization of the capsule network architecture

Preliminary experiments with the SegCaps architecture [16] revealed severe weaknesses. As also observed in [3], SegCaps was not able to produce reasonable results when used for multi-class segmentation. We thus forewent investigating this architecture any further and completely focused on Matwo-CapsNet.

So far, the performance of Matwo-CapsNet has only been demonstrated for chest X-ray as well as computed tomography images. These modalities are very different from ultrasound in terms of texture and noise structure. Ultrasound images are typically governed by speckle noise which tends to make borders between different tissues rather unclear and harder to detect. Furthermore, parts of the images are often obscured by shadow artifacts leading to a local reduction of information. We can thus assume that Matwo-CapsNet’s hyperparameters have to be tuned in order to optimize the network structure toward IVUS image segmentation. This procedure was performed on the 20 MHz dataset.

As already mentioned in the previous section the following structural parameters play an important role in Matwo-CapsNet and have been investigated regarding their impact on the IVUS segmentation results:

- Treatment of the pose matrix
- Routing type and number of routing iterations
- Performing a reconstruction regularization
- Window size of locally constrained routing
- Pose matrix shape
- Appearance matrix shape
- Number of capsule types throughout the network

### Comparison between capsule network and U-Net Res

We compared our tuned capsule network with a state-of-the-art encoder–decoder CNN similar to the U-Net [20] but built with residual blocks [8] analogous to [18]. We call it U-Net Res throughout this work. Both the baseline CNN and the capsule network had an equal number of parameters. We chose a U-Net-like baseline CNN due to two reasons. First, previous work reports state-of-the-art results using encoder-decoder CNNs [7, 15, 17, 19, 28, 29, 31]. Second, the capsule network also features an encoder–decoder structure which makes both networks more comparable. We furthermore studied how both networks behave when the number of parameters is reduced. Small networks with less parameters are of great importance when it comes to running these on embedded systems or mobile devices [6], because here the amount of available memory is usually rather limited.

We used the 20 MHz dataset and training set sizes of 250, 150 and only 50 training images and investigated which of the networks were able to cope better with smaller datasets. Networks which generally perform better on such small datasets are advantageous for medical image datasets, particularly for few-shot learning tasks [26]. In addition, we evaluated our approach on the 40 MHz dataset in order to investigate whether the capsule architecture optimized for the 20 MHz dataset could readily be used for slightly different data.

### Training and evaluation

Preliminary experiments showed that Matwo-CapsNet performed best with the spread loss, which was introduced specifically for capsule networks [22]. The U-Net Res on the other hand performed best with the generalized Dice loss, a state-of-the-art loss function for medical image segmentation [24]. We therefore used the spread loss for all capsule networks and the generalized Dice loss for all U-Net Res.

**Fig. 1:**
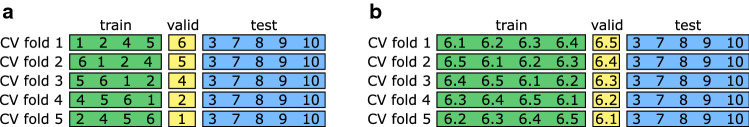
Overview of the used CV schemes and the distribution of patients among the individual sets. **a** CV scheme for scenarios one (250 training images) and two (150 training images). Scenario two only uses 60% of images from every patient. **b** CV scheme for scenario three (50 training images). All images in the training and validation sets originate solely from patient six

**Table 1:** Segmentation performances as a function of different treatments of the pose (or pose transformation) matrix

Pose matrix treatment	Dice coefficient		Ave. Hausdorff distance [px]	
	Vessel wall	Lumen	Vessel wall	Lumen
Normalized transf. w/coord. add.	$66.27 \pm 2.77$	$90.50 \pm 1.34$	$2.73 \pm 0.08$	$1.27 \pm 0.86$
Normalized w/ coord. add.	$\mathbf{79}.11 \pm \mathbf{1}.15$	$\mathbf{93}.52 \pm \mathbf{1}.05$	$\mathbf{1}.37 \pm \mathbf{0}.24$	$\mathbf{0}.66 \pm \mathbf{0}.70$
Normalized w/o coord. add.	$69.98 \pm 2.69$	$90.15 \pm 0.88$	$3.11 \pm 0.85$	$1.38 \pm 0.50$
No modifications	$68.3 \pm 2.07$	$89.31 \pm 0.74$	$3.06 \pm 0.59$	$1.64 \pm 0.55$

Bold values indicate best results

**Table 2:** Segmentation performances as a function of different routing algorithms performed with three routing iterations

Routing algorithm	Dice coefficient		Ave. Hausdorff distance [px]	
	Vessel wall	Lumen	Vessel wall	Lumen
Dynamic routing	$73.14 \pm 1.23$	$90.89 \pm 0.78$	$2.13 \pm 0.43$	$0.96 \pm 0.44$
Dual routing	$\mathbf{79}.11 \pm \mathbf{1}.15$	$\mathbf{93}.52 \pm \mathbf{1}.05$	$\mathbf{1}.37 \pm \mathbf{0}.24$	$\mathbf{0}.66 \pm \mathbf{0}.70$

Bold values indicate best results

**Table 3:** Segmentation performances as a function of different numbers of routing iterations performed with dual routing

# Iterations	Dice coefficient		Ave. Hausdorff distance [px]	
	Vessel wall	Lumen	Vessel wall	Lumen
1	$55.06 \pm 1.60$	$86.23 \pm 0.79$	$6.13 \pm 0.84$	$2.89 \pm 0.25$
2	$76.36 \pm 0.77$	$91.71 \pm 1.07$	$1.73 \pm 0.17$	$0.70 \pm 0.32$
3	$\mathbf{79}.11 \pm \mathbf{1}.15$	$\mathbf{93}.52 \pm \mathbf{1}.05$	$\mathbf{1}.37 \pm \mathbf{0}.24$	$\mathbf{0}.66 \pm \mathbf{0}.70$
4	$74.48 \pm 2.61$	$91.49 \pm 1.27$	$1.95 \pm 0.14$	$0.89 \pm 0.59$
5	$67.77 \pm 3.78$	$90.18 \pm 1.57$	$2.29 \pm 0.44$	$1.10 \pm 0.44$

Bold values indicate best results

We carried out fivefold cross-validation (CV) for all experiments in order to get meaningful statistics. We investigated three different training set sizes of the 20 MHz dataset:

1. 250 training images: every CV-fold comprised 50 images of a single patient, resulting in five different patients in the training set. The remaining 185 images of the dataset, again from five different patients, were used for testing.
2. 150 training images: same as (1) but with only 30 images per patient in the CV-folds. Same test set as (1).
3. 50 training images: only data of a single patient divided into CV-folds of ten images. This setting makes it difficult for networks to generalize to the unseen test data because the validation sets highly correlate with the training sets. Same test set as (1) and (2).

A detailed overview of the CV schemes is depicted in Fig. 1. All images were resized to 256 $\times$ 256 pixels and augmented by random rotations and flips on-the-fly during training. As evaluation metrics we chose the Dice coefficient as a measure of overlap and the average Hausdorff distance [5] as a measure of edge alignment between the predicted and ground-truth segmentation masks. The average Hausdorff distance between two sets *A* and *B* is defined as

$$\begin{aligned} d_H^{ave} = \max \left\{ \mathop {\text {mean}}\limits _{a\in A}\min _{b\in B}d(a,b),\quad \mathop {\text {mean}}\limits _{b\in B}\min _{a\in A}d(a,b)\right\} \end{aligned}$$

with the Euclidean distance $d(\cdot ,\cdot )$. Due to the mean operations, $d_H^{ave}$ is less sensitive to outliers [5, 25] which makes comparing segmentation pixel masks more meaningful than using the ordinary Hausdorff distance. The average Hausdorff distance is therefore quite similar to the average symmetric surface distance which computes the mean instead of the max of both directed distances. For completeness and comparability to previous work, we do also report the ordinary Hausdorff distance.

All networks were trained with the Adam optimizer. Via preliminary grid-searching, we found a learning rate of $\ell =1e-3$ to be optimal for the Matwo-CapsNet, whereas it was $\ell =2e-4$ for the U-Net Res. We trained every network for 200 epochs and validated after every epoch with the validation set by computing Dice coefficients. After training, the model which performed best on the validation set was chosen to be evaluated with the test set.

Additionally, we evaluated our approach on the 40 MHz dataset. Due to its small size of 77 images, we only evaluated a single training set size. We performed fivefold cross-validation with ten images per fold and 27 images in the test set. All other settings were the same as above.

## Results and discussion

### Optimization of the capsule network architecture

Grid-searching all possible architecture hyperparameters was not feasible regarding temporal and computing resources. We thus used a partially greedy approach starting with a set of parameters used in the original Matwo-CapsNet paper [3]. However, we changed the numbers of capsule types in the encoding path (network shape) from {5,5,6,7} to {3,5,7,9} and used two convolutional capsule layers per level. The order of the numbers of capsule types in the decoding path is vice versa. The initial shape of the pose matrix was 4 $\times$ 4, whereas the appearance matrix had a shape of 5 $\times$ 5. If improvements were found, these were integrated into the network. Exceptions are mentioned in the text. For the sake of clarity, we used only the average Hausdorff distance measured in pixels as the basis for evaluation in this section, in addition to the Dice coefficient.

First, we investigated how different treatments of the pose matrix affected the segmentation performance. Originally, Hinton et al. [10] did not normalize the pose matrix but proposed to add scaled coordinates to the last matrix column relative to the center of the capsule’s receptive field. Bonheur et al. [3] introduced the idea of normalizing every column of the pose transformation matrix such that these have unit length. We compared this method with three other ones: normalizing the pose matrix with subsequent addition of scaled coordinates, normalizing the pose matrix without adding scaled coordinates and no manipulation at all. The corresponding results are given in Table 1. We can see that the approach of normalizing the pose matrix with subsequent scaled coordinate addition led to the best segmentation performance by far.

**Table 4:** Segmentation performances as a function of different approaches to adding a reconstruction regularization. The underlying network shape was {3,5,7,9}

Reconstruction	Dice coefficient		Ave. Hausdorff distance [px]	
	Vessel wall	Lumen	Vessel wall	Lumen
Without	$\mathbf{79}.11 \pm \mathbf{1}.15$	$\mathbf{93}.52 \pm \mathbf{1}.05$	$\mathbf{1}.37 \pm \mathbf{0}.24$	$\mathbf{0}.66 \pm \mathbf{0}.70$
From all classes	$77.62 \pm 1.39$	$92.23 \pm 1.39$	$1.60 \pm 0.28$	$0.71 \pm 0.37$
From pos. classes	$76.37 \pm 2.10$	$92.52 \pm 1.05$	$1.65 \pm 0.15$	$0.82 \pm 0.67$
From lowest level	$76.60 \pm 1.56$	$92.49 \pm 0.78$	$1.60 \pm 0.26$	$0.77 \pm 0.60$

Bold values indicate best results

**Table 5:** Segmentation performances as a function of different pose matrix sizes obtained with a network of shape {3,5,7,9} and an appearance matrix with shape 5 $\times$ 5

Pose matrix shape	Dice coefficient		Ave. Hausdorff distance [px]	
	Vessel wall	Lumen	Vessel wall	Lumen
$2\times 2$	$37.82 \pm 29.02$	$58.19 \pm 24.43$	$24.12 \pm 26.14$	$26.67 \pm 31.33$
$3\times 3$	$75.71 \pm 1.84$	$91.33 \pm 2.07$	$1.73 \pm 0.23$	$0.79 \pm 0.60$
$4\times 4$	$\mathbf{79}.11 \pm \mathbf{1}.15$	$\mathbf{93}.52 \pm \mathbf{1}.05$	$\mathbf{1}.37 \pm \mathbf{0}.24$	$\mathbf{0}.66 \pm \mathbf{0}.70$
$5\times 5$	$77.16 \pm 1.44$	$92.76 \pm 0.65$	$1.71 \pm 0.24$	$0.76 \pm 0.52$

Bold values indicate best results

**Table 6:** Segmentation performances as a function of different appearance matrix sizes obtained with a network of shape {3,5,7,9} and a pose matrix with shape 4 $\times$ 4

Appearance matrix shape	Dice coefficient		Ave. Hausdorff distance [px]	
	Vessel wall	Lumen	Vessel wall	Lumen
$2\times 2$	$75.60 \pm 1.87$	$91.22 \pm 1.45$	$1.67 \pm 0.06$	$0.81 \pm 0.44$
$3\times 3$	$76.94 \pm 1.24$	$92.45 \pm 1.14$	$1.75 \pm 0.40$	$0.82 \pm 0.60$
$4\times 4$	$76.65 \pm 0.52$	$92.64 \pm 0.69$	$1.59 \pm 0.18$	$0.64 \pm 0.43$
$5\times 5$	$\mathbf{79}.11 \pm \mathbf{1}.15$	$\mathbf{93}.52 \pm \mathbf{1}.05$	$\mathbf{1}.37 \pm \mathbf{0}.24$	$0.66 \pm 0.70$
$6\times 6$	$76.07 \pm 1.62$	$92.18 \pm 1.25$	$1.78 \pm 0.19$	$\mathbf{0}.62 \pm \mathbf{0}.14$

Bold values indicate best results

**Table 7:** Segmentation performances as a function of network depth and the number of capsule types per level

Network shape	Dice coefficient		Ave. Hausdorff distance [px]	
	Vessel wall	Lumen	Vessel wall	Lumen
{3,4,5,6}	$75.63 \pm 2.20$	$91.94 \pm 0.29$	$1.80 \pm 0.19$	$0.66 \pm 0.12$
{3,4,5,6,7}	$\mathbf{76}.39 \pm \mathbf{1}.23$	$\mathbf{92}.94 \pm \mathbf{0}.42$	$\mathbf{1}.56 \pm \mathbf{0}.26$	$\mathbf{0}.43 \pm \mathbf{0}.06$
{3,5,7,9}	$\mathbf{79}.11 \pm \mathbf{1}.15$	$\mathbf{93}.52 \pm \mathbf{1}.05$	$\mathbf{1}.37 \pm \mathbf{0}.24$	$0.66 \pm 0.70$
{3,5,7,9,11}	$78.97 \pm 0.67$	$93.33 \pm 0.60$	$1.30 \pm 0.20$	$\mathbf{0}.46 \pm \mathbf{0}.13$
{3,6,12}	$74.38 \pm 2.10$	$90.54 \pm 1.05$	$1.73 \pm 0.19$	$1.14 \pm 0.46$
{3,6,12,24}	$79.98 \pm 0.73$	$92.99 \pm 0.82$	$1.32 \pm 0.25$	$0.51 \pm 0.26$
{3,6,12,24,48}	$\mathbf{81}.16 \pm \mathbf{1}.88$	$\mathbf{94}.59 \pm \mathbf{0}.38$	$\mathbf{1}.02 \pm \mathbf{0}.30$	$\mathbf {037 \pm 0.70}$

Bold values indicate best results

We then investigated how the results were affected by using either dual routing or dynamic routing as well as the number of routing iterations. Tables 2 and 3 show that using dual routing with three routing iterations performed best. This means that treating appearance and pose features separately is also beneficial for IVUS segmentation. Increasing the number of routing iterations to values higher than three leads to a decrease in segmentation performance, a tendency also shown in [10] for classification. Due to the larger number of routing iterations, the capacity of the network increases, which eventually leads to overfitting.

**Table 8:** Segmentation performances as a function of different window sizes for locally constrained routing obtained with a network of shape {3,6,12,24}

Window size	Dice coefficient		ave. Hausdorff distance [px]	
	Vessel wall	Lumen	Vessel wall	Lumen
$3\times 3$	$77.25 \pm 1.03$	$92.42 \pm 1.34$	$1.63 \pm 0.13$	$0.80 \pm 0.58$
$5\times 5$	$79.78 \pm 2.03$	$\mathbf{94}.40 \pm \mathbf{0}.40$	$1.27 \pm 0.41$	$\mathbf{0}.38 \pm \mathbf{0}.26$
$7\times 7$	$\mathbf{80}.46 \pm \mathbf{1}.48$	$93.40 \pm 0.95$	$\mathbf{1}.24 \pm \mathbf{0}.22$	$0.44 \pm 0.16$

Bold values indicate best results

**Fig. 2:**
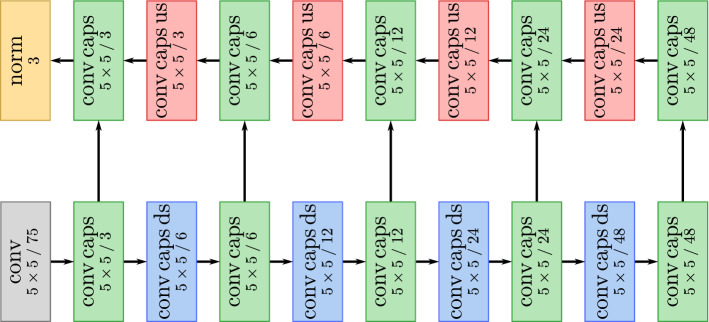
Sketch of the optimized capsule network architecture. The ordinary convolutional layer is colored gray. Convolutional capsule layers (with downsampling/upsampling) are colored green (blue/red). The digits indicate window size as well as the number of capsule types (feature maps) after convolutional capsule layers (convolutional layers). The last layer computes the Frobenius norm of pose matrix and appearance matrix and multiplies both resulting values for each capsule (i.e., pixel) and all three segmentation classes

**Table 9:** Segmentation performances of capsule networks and baseline U-Net Res for different sizes of the 20 MHz dataset measured by Dice coefficient

# Images	# Params	Network	Dice coefficient	
			Vessel wall	Lumen
50	32 k	CapsNet	$\mathbf{71}.05 \pm \mathbf{2}.25$	$\mathbf{90}.63 \pm \mathbf{1}.06$
		U-Net Res	$59.42 \pm 5.98$	$78.60 \pm 9.20$
	102 k	CapsNet	$\mathbf{72}.00 \pm \mathbf{2}.71$	$\mathbf{91}.73 \pm \mathbf{0}.79$
		U-Net Res	$61.31 \pm 4.77$	$76.07 \pm 5.23$
	420 k	CapsNet	$\mathbf{73}.99 \pm \mathbf{1}.55$	$\mathbf{91}.58 \pm \mathbf{0}.78$
		U-Net Res	$68.71 \pm 2.09$	$87.04 \pm 2.45$
150	32 k	CapsNet	$\mathbf{77}.17 \pm \mathbf{0}.92$	$\mathbf{93}.00 \pm \mathbf{0}.40$
		U-Net Res	$73.17 \pm 1.55$	$90.28 \pm 1.33$
	102 k	CapsNet	$\mathbf{78}.74 \pm \mathbf{1}.02$	$\mathbf{93}.22 \pm \mathbf{1}.01$
		U-Net Res	$75.12 \pm 1.83$	$90.58 \pm 0.95$
	420 k	CapsNet	$\mathbf{79}.07 \pm \mathbf{1}.40$	$\mathbf{93}.84 \pm \mathbf{0}.33$
		U-Net Res	$78.92 \pm 1.48$	$93.38 \pm 0.83$
250	32 k	CapsNet	$\mathbf{79}.11 \pm \mathbf{1}.15$	$\mathbf{93}.52 \pm \mathbf{1}.05$
		U-Net Res	$75.79 \pm 2.11$	$92.07 \pm 2.86$
	102 k	CapsNet	$\mathbf{79}.98 \pm \mathbf{0}.73$	$\mathbf{92}.99 \pm \mathbf{0}.82$
		U-Net Res	$76.67 \pm 2.26$	$92.24 \pm 1.21$
	420 k	CapsNet	$\mathbf{81}.16 \pm \mathbf{1}.88$	$\mathbf{94}.59 \pm \mathbf{0}.38$
		U-Net Res	$80.15 \pm 1.35$	$94.21 \pm 0.76$

Bold values indicate best results

The resulting segmentation performance when using three different approaches for reconstruction as a regularization method is shown in Table 4. First, the reconstruction was performed from the capsules belonging to all classes of the last layer. Second, only the capsules from the positive classes of the last layer were used. And third, the capsules of the lowest network level were used. We found no performance improvement through adding a reconstruction regularization. Additionally, incorporating a reconstruction heavily increased training time and VRAM load. We therefore refrained from using a reconstruction just like Bonheur et al. [3].

**Table 10:** Segmentation performances of capsule networks and baseline U-Net Res for different sizes of the 20 MHz dataset measured by ordinary and average Hausdorff distance

# Images	# Params	Network	Hausdorff distance [mm]		Ave. Hausdorff dist. [mm]	
			Vessel wall	Lumen	Vessel wall	Lumen
50	32 k	CapsNet	$\mathbf {.521 \pm .059}$	$\mathbf {.355 \pm .040}$	$\mathbf {.060 \pm .009}$	$\mathbf {.022 \pm .003}$
		U-Net Res	$.825 \pm .116$	$.629 \pm .123$	$.158 \pm .059$	$.097 \pm .050$
	102 k	CapsNet	$\mathbf {.570 \pm .042}$	$\mathbf {.353 \pm .056}$	$\mathbf {.062 \pm .010}$	$\mathbf {.016 \pm .003}$
		U-Net Res	$.953 \pm .164$	$.633 \pm .090$	$.203 \pm .060$	$.126 \pm .045$
	420 k	CapsNet	$\mathbf {.419 \pm .040}$	$\mathbf {.297 \pm .020}$	$\mathbf {.046 \pm .005}$	$\mathbf {.017 \pm .002}$
		U-Net Res	$.627 \pm .084$	$.378 \pm .055$	$.086 \pm .019$	$.053 \pm .020$
150	32 k	CapsNet	$\mathbf {.393 \pm .033}$	$\mathbf {.252 \pm .026}$	$\mathbf {.036 \pm .003}$	$\mathbf {.013 \pm .002}$
		U-Net Res	$.592 \pm .082$	$.427 \pm .087$	$.066 \pm .007$	$.032 \pm .010$
	102 k	CapsNet	$\mathbf {.406 \pm .049}$	$\mathbf {.261 \pm .015}$	$\mathbf {.036 \pm .008}$	$\mathbf {.014 \pm .003}$
		U-Net Res	$.518 \pm .076$	$.365 \pm .079$	$.055 \pm .013$	$.028 \pm .007$
	420 k	CapsNet	$\mathbf {.352 \pm .035}$	$\mathbf {.243 \pm .038}$	$\mathbf {.035 \pm .004}$	$\mathbf {.013 \pm .005}$
		U-Net Res	$.416 \pm .139$	$.265 \pm .067$	$.038 \pm .010$	$.015 \pm .011$
250	32 k	CapsNet	$\mathbf {.470 \pm .067}$	$\mathbf {.273 \pm .091}$	$\mathbf {.036 \pm .006}$	$\mathbf {.017 \pm .016}$
		U - Net Res	$.625 \pm .174$	$.507 \pm .233$	$.060 \pm .019$	$.049 \pm .064$
	102 k	CapsNet	$\mathbf {.394 \pm .072}$	$\mathbf {.234 \pm .038}$	$\mathbf {.033 \pm .010}$	$\mathbf {.010 \pm .006}$
		U-Net Res	$.643 \pm .175$	$.342 \pm .095$	$.065 \pm .030$	$.028 \pm .019$
	420 k	CapsNet	$\mathbf {.313 \pm .052}$	$.207 \pm .048$	$\mathbf {.027 \pm .007}$	$.010 \pm .006$
		U-Net Res	$.353 \pm .063$	$\mathbf {.196 \pm .033}$	$.031 \pm .005$	$\mathbf {.008 \pm .002}$

Bold values indicate best results

We then investigated different sizes of the pose and appearance matrix. Tables 5 and 6 show the corresponding results. Using a pose matrix with shape 4 $\times$ 4 and an appearance matrix with shape 5 $\times$ 5 led to the best results. Interestingly, the performance drops when choosing the larger matrix sizes.

Regarding the underlying encoder–decoder architecture, we investigated how different network depths (and thus different numbers of downsamplings) affect the segmentation performance. Furthermore, we compared different alternatives for increasing the number of capsule types in the encoding path by either adding a fixed number of capsule types or doubling these in each level. The approaches in [3, 16] are non-doubling (likely due to limitations of computational resources) but Table 7 shows that doubling is rather beneficial when performed along with increasing the depth to five levels.

The window size for locally constrained routing is an important hyperparameter because it drastically affects the number of weights and the size of the capsules’ receptive fields. Table 8 depicts the segmentation performances with different window sizes. Due to limitations with respect to computational resources, we were not able to apply window sizes of 7 $\times$ 7 to networks with shape {3,6,12,24,48}. We therefore used a network with shape {3,6,12,24} for this comparison. We do not see clear improvements when switching from $5\times 5$ to $7\times 7$ windows. We therefore stuck to a window size of $5\times 5$ for further experiments which is the same as in [3, 16].

**Fig. 3:**
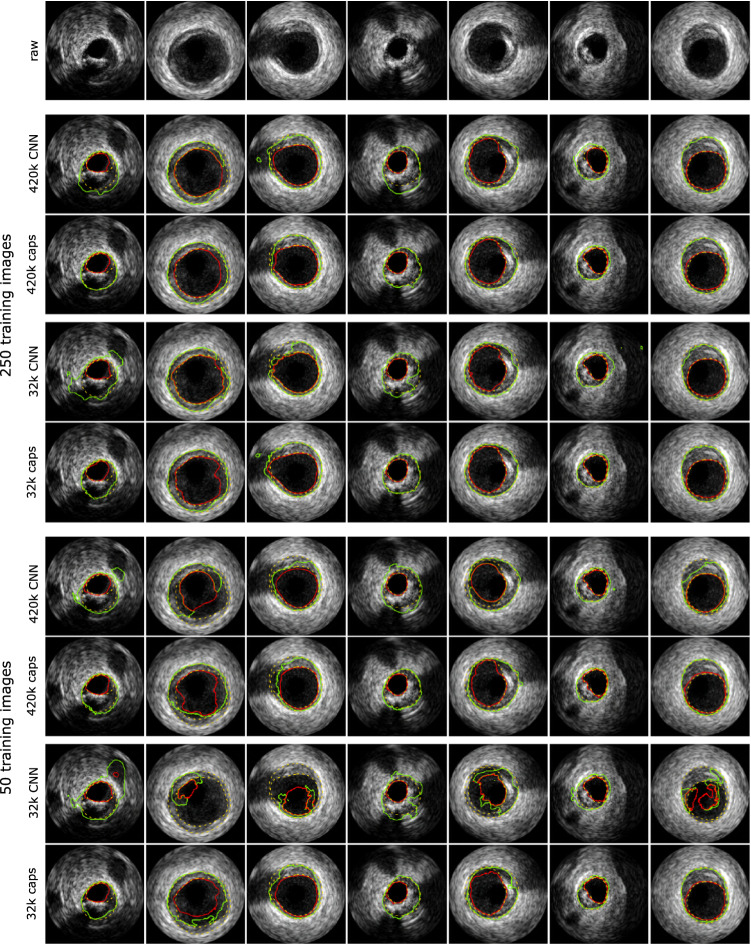
Comparison of exemplary segmentation results between capsule networks and CNNs for the 20 MHz dataset. Shown are predictions of large networks with 420 k parameters and small networks with 32 k parameters. Ground truth annotations of lumen border and external elastic membrane are depicted with yellow dashed lines. The predicted contours with red and green solid lines, respectively

The structural parameters of Matwo-CapsNet which led to the best segmentation performance are as follows:

- Normalizing pose matrix and adding scaled coordinates
- Dual routing with three iterations
- No reconstruction
- Routing window size: 5 $\times$ 5
- Pose matrix shape: 4 $\times$ 4
- Appearance matrix shape: 5 $\times$ 5
- Network shape: {3, 6, 12, 24, 48}

The resulting architecture differs from the original Matwo-CapsNet architecture proposed in [3]. The major differences are the treatment of the pose matrix (normalizing the pose matrix instead of the pose transformation matrix), the increased network depth of five levels and the doubling of capsule types at each level leading to 48 capsule types at the lowest level. Making the network deeper while only adding a fixed amount of capsule types per level increased the performance substantially less or even led to performance drops. Figure 2 depicts a sketch of the optimized capsule network architecture.

### Comparison between capsule network and U-Net Res

The resulting segmentation performances on the 20 MHz dataset are given in Tables 9 and 10. One can clearly see the tendency of the capsule network to outperform the U-Net Res when the training sets get smaller as well as when the network sizes decrease. We can thus deduce that developing part-whole relationships in capsule networks is beneficial for the segmentation of ultrasound images when dealing with data scarcity or small networks.

**Table 11:** Segmentation performances of capsule networks and baseline U-Net Res on the 40 MHz dataset measured by Dice coefficient

# Params	Network	Dice coefficient	
		Vessel wall	Lumen
32 k	CapsNet	$\mathbf{66}.57 \pm \mathbf{2}.17$	$\mathbf{88}.85 \pm \mathbf{0}.84$
	U-Net Res	$58.89 \pm 0.44$	$86.90 \pm 0.70$
102 k	CapsNet	$\mathbf{67}.99 \pm \mathbf{1}.88$	$\mathbf{88}.50 \pm \mathbf{0}.53$
	U-Net Res	$61.64 \pm 1.60$	$85.42 \pm 0.68$
420 k	CapsNet	$\mathbf{73}.09 \pm \mathbf{1}.54$	$\mathbf{90}.84 \pm \mathbf{0}.68$
	U-Net Res	$70.26 \pm 2.00$	$90.57 \pm 0.36$

Bold values indicate best results

**Table 12:** Segmentation performances of capsule networks and baseline U-Net Res on the 40 MHz dataset measured by ordinary and average Hausdorff distance

# Params	Network	Hausdorff distance [mm]		Ave. Hausdorff dist. [mm]	
		Vessel wall	Lumen	Vessel wall	Lumen
32 k	CapsNet	$\mathbf {1.115 \pm .073}$	$\mathbf {.632 \pm .047}$	$\mathbf {.121 \pm .009}$	$\mathbf {.034 \pm .003}$
	U-Net Res	$1.599 \pm .177$	$.995 \pm .047$	$.198 \pm .019$	$.065 \pm .008$
102 k	CapsNet	$\mathbf {.874 \pm .097}$	$\mathbf {.514 \pm .096}$	$\mathbf {.087 \pm .008}$	$\mathbf {.025 \pm .005}$
	U-Net Res	$1.286 \pm .106$	$1.652 \pm .110$	$.162 \pm .018$	$.078 \pm .026$
420 k	CapsNet	$\mathbf {.857 \pm .063}$	$\mathbf {.463 \pm .048}$	$\mathbf {.085 \pm .006}$	$\mathbf {.022 \pm .006}$
	U-Net Res	$.996 \pm .139$	$.522 \pm .065$	$.097 \pm .017$	$.028 \pm .006$

Bold values indicate best results

**Fig. 4:**
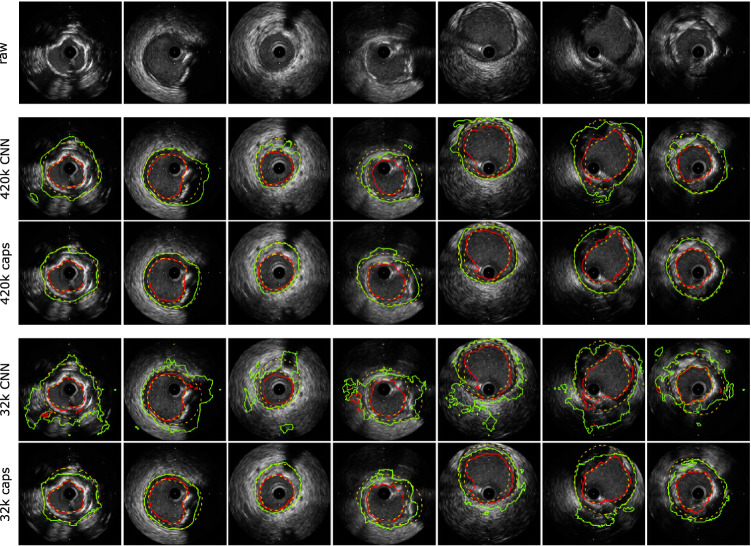
Comparison of exemplary segmentation results between capsule networks and CNNs for the 40 MHz dataset. Shown are predictions of large networks with 420 k parameters and small networks with 32 k parameters. Ground truth annotations of lumen border and external elastic membrane are depicted with yellow dashed lines. The predicted contours with red and green solid lines, respectively

For vessel wall segmentation with 250 training images, the relative improvement regarding the Dice coefficient is 1.3% in the case of networks with 420 k parameters and increases to 4.5% for networks with 32 k parameters. The corresponding improvements of the average Hausdorff distance are 12.8% and 46.1%. When using 50 training images, the improvements of the Dice score are 4.6% for networks with 420 k parameters and 19.6% for networks with 32 k parameters. The corresponding average Hausdorff distances improve by 26.5% and 61.9%.

Furthermore, we see that the performance drops of the capsule networks, when decreasing the number of parameters, are substantially smaller compared to the baseline CNNs. In the case of the vessel wall, the Dice scores drop about − 2.4% vs. − 5.8% for networks trained with 250 images and − 1.2% vs. − 15.6% for networks trained with 50 images.

Figure 3 shows exemplary segmentation results for the cases of 250 and 50 training images. It can be seen that the capsule networks are able to complete the vessel wall shape in shadowed regions quite well (see, e.g., Fig. 3 columns 1, 3 and 4), whereas the CNNs fail to do so. Additionally, the predictions of the capsule networks always exhibit a closed vessel wall shape which completely surrounds the lumen. This is not always the case for the CNN predictions (see Fig. 3 columns 2, 3 and 5). Hence, we can assume that the capsule network learned some kind of shape representation of vessel walls and is able to interpolate missing grayvalue gradient information.

In addition, we provide segmentation results for the 40 MHz dataset in Tables 11 and 12. The picture is generally the same as for the 20 MHz dataset. Exemplary segmentation results are depicted in Fig. 4. It can be seen that the capsule network is capable of inferring vessel borders in shadowed regions, as was the case for 20 MHz images. Furthermore, the decrease in performance when reducing the number of network parameters is substantially smaller compared to the baseline CNN. All in all, this shows that the capsule network architecture optimized for the 20 MHz dataset can be readily used for the 40 MHz dataset.

The major drawback of the capsule network is the long training time compared to the U-Net Res. The largest capsule network needed approximately 16 h training time for five-fold cross-validation, whereas training the corresponding U-Net Res only took roughly 45 min. Also the required amount of graphics memory differed largely. The largest U-Net Res model needed about 3.5 GB of VRAM, whereas the largest capsule network occupied about 20 GB. All experiments were performed on an NVIDIA Titan RTX GPU with 24 GB of VRAM. The main reason for this large difference is the iterative routing process. This also affects the inference time which was more than 30 times longer than the corresponding CNN inference time (e.g., 100 ms vs. 3 ms for networks with 420 k parameters).

Nevertheless, in the case of IVUS, image segmentation capsule networks turned out to be quite performant on small datasets, even with a rather small network size of 32 k parameters. This makes capsule networks promising candidates for few-shot learning tasks like patient adaptation or detection of diseases with small prevalence as well as for applications on mobile devices.

## Conclusion

We systematically optimized a capsule network architecture for segmentation of intravascular ultrasound (IVUS) images. The approach of doubling the number of capsule types at each downsampling level analogous to typical CNN architectures turned out to be quite beneficial. We showed that our capsule network performs particularly well on a small dataset compared to a corresponding U-Net Res. We thus assume that capsule networks are promising candidates for ultrasound image segmentation in general when dealing with data scarcity. This could make capsule networks suitable for few- or even single-shot learning tasks as well as applications for mobile devices. Further research should focus on tackling such tasks with capsule networks.
